# Air Pollution—An Overlooked Risk Factor for Idiopathic Pulmonary Fibrosis

**DOI:** 10.3390/jcm10010077

**Published:** 2020-12-28

**Authors:** Sebastian Majewski, Wojciech J. Piotrowski

**Affiliations:** Department of Pneumology and Allergy, Medical University of Lodz, 90-153 Lodz, Poland; wojciech.piotrowski@umed.lodz.pl

**Keywords:** idiopathic pulmonary fibrosis, IPF, air pollution, risk factors, health outcomes

## Abstract

Air pollution is a major environmental risk to health and a global public health concern. In 2016, according to the World Health Organization (WHO), ambient air pollution in cities and rural areas was estimated to cause 4.2 million premature deaths. It is estimated that around 91% of the world’s population lives in places where air pollution exceeds the limits recommended by the WHO. Sources of air pollution are multiple and context-specific. Air pollution exposures are established risk factors for development and adverse health outcomes in many respiratory diseases, including asthma, chronic obstructive pulmonary disease (COPD), or lung cancer. However, possible associations between air pollution and idiopathic pulmonary fibrosis (IPF) have not been adequately studied and air pollution seems to be an underrecognized risk factor for IPF. This narrative review describes potential mechanisms triggered by ambient air pollution and their possible roles in the initiation of the pathogenic process and adverse health effects in IPF. Additionally, we summarize the most current research evidence from the clinical studies supporting links between air pollution and IPF.

## 1. Introduction

Clean air is a basic requirement for human health and well-being. According to the World Health Organization (WHO), air pollution kills an estimated seven million people worldwide every year. The WHO data shows that 9 out of 10 people breathe air that exceeds WHO guideline limits containing high levels of pollutants, with low- and middle-income countries suffering from the highest exposures [1]. Human airway epithelium constitutes the first line of interaction with an atmospheric environment. This interaction between the airway epithelium and the inhaled environment is crucial in the pathobiology of respiratory diseases [2]. Idiopathic pulmonary fibrosis (IPF) represents one of the common types of interstitial lung diseases (ILDs), a heterogeneous group of chronic lung conditions characterized by inflammation and fibrosis of the pulmonary parenchyma. The natural history of IPF is outlined by inevitable progressive decline of lung function, physical activity limitation, impairment of quality of life, and premature death, with a median survival of 3 to 5 years [3]. The chronic, repetitive, subclinical micro-injuries to the alveolar epithelium caused by the exposure to various noxious stimuli are leading in the predisposed individuals to subsequent dysfunction of the alveolar epithelium, which is central for the initiation and perpetuation of the pathogenic process in IPF [3]. Both ambient and indoor air pollution exposures are established risk factors for respiratory diseases [4]. Moreover, air pollution leads to a number of adverse health outcomes in asthma, chronic obstructive pulmonary disease (COPD), respiratory infections, lung cancer, and lung transplant recipients [5,6]. Yet, relatively little attention has been given to investigate the relationship between air pollution and IPF. Moreover, ambient air pollution exposure seems to be often overlooked as an important risk factor for disease initiation and adverse respiratory outcomes in IPF. This narrative review aims to examine the current concepts on the potential mechanisms by which air pollution triggers and/or worsens IPF and update on actual research evidence on the health effects of ambient air pollutants in IPF. For the purpose of the latter, we performed a literature search using the MEDLINE database to identify studies linking air pollution with IPF. A combination of the following keywords was used: “air pollution”, “outdoor pollution”, “atmospheric pollution”, “particulate matter”, “PM”, “ozone” “idiopathic pulmonary fibrosis”, “IPF”, “interstitial lung abnormalities”, “interstitial lung disease”. Studies included in our review had to be clinical, original research studies, of prospective or retrospective design, published in English within the last 10 years, and tackling the problem of interest. After careful selection, we identified a total of 10 studies considered relevant for the scope of our topic, and review their findings in a descriptive manner below.

## 2. Types and Sources of Air Pollutants and Potential Mechanisms Triggered by Air Pollutants Affecting the Risk and Health Outcomes in IPF

### 2.1. Types and Sources of Air Pollutants

The term “air pollution” refers to a complex mix of gases and particles. The most important gaseous compounds are nitric dioxide (NO_2_), other nitrogen oxides (NO_x_), carbon monoxide (CO), sulfur dioxide (SO_2_), and ozone (O_3_). Particulate matter (PM) is classified according to its aerodynamic diameter to coarse particles, PM_10_ (≤10 µm), fine particles, PM_2.5_ (≤2.5 µm), and ultrafine particles PM_0.1_ (≤0.1 µm). Industry, primarily based on coal burning, was the main source of air pollution in the past. Today, still, industrial pollution and so-called traffic-related air pollution (TRAP) are the main sources of ambient air pollution. PM is generated through two main processes: natural (which includes phenomena like sea sprays, volcanic eruptions, forest fires, and soil erosion) and anthropogenic, related to road traffic and other forms of transportation, and industry (mainly electricity generation, mining, welding, or building) [7]. The burning of fuels, such as diesel, petrol, oil, wood, is related to PM production. When particles are generated and released directly to the atmosphere, they are called primary PM. Secondary PM is generated due to chemical reactions, gas-to-particle conversion processes, which include nucleation, condensation, and coagulation of gases and particles, leading to the increase in their diameter [7,8]. The particle diameter is crucial for deposition in the airways. Gases and particles that form the aerosol deposit in the airways through a combination of inertial impaction, gravitational sedimentation, and Brownian diffusion. The coarse fraction (PM_10-2.5_) settles in the upper airways. Fine fraction (PM_2.5_) is deposited in the lungs, and smaller particles may be transported through the capillaries to the systemic circulation, and further to other organs of the body. The large surface area of PM contributes to the combination of toxic compounds, including polycyclic aromatic hydrocarbons (PAHs), volatile organic compounds (VOCs), and transition metals, which are responsible for free-radicals formation [7,8].

Smog is a term used for the description of a mixture of pollutant gases and particles in the atmosphere, forming intense air pollution. The term was coined from two words, smoke and fog, and refers to the situation when air pollution episodes lead to a decrease in air transparency. Photochemical smog is the smog that contains a high concentration of O_3_ and other reactive chemical compounds generated by the action of sunlight on NO_2_ and hydrocarbons. It is considered especially toxic and is mainly related to road traffic air pollution in the summer months. Stagnant air, windless weather, and specific terrain configuration (river valleys) have been assigned as main causative factors of tragic environmental pollution episodes leading to thousands of excess deaths in Meuse Valley, Belgium (1930), Donora, Pennsylvania, USA (1948), and London, United Kingdom (1952) [9].

### 2.2. Potential Mechanisms Triggered by Air Pollutants Affecting the Risk and Health Outcomes in IPF

**Figure 1:**
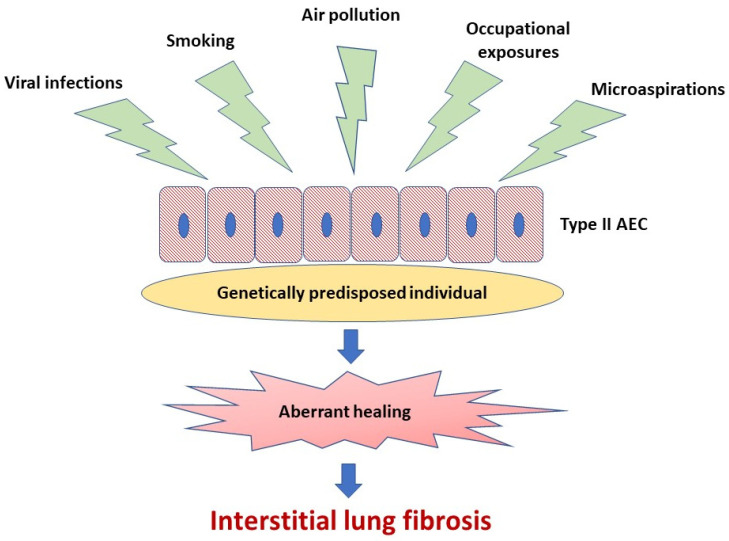
Idiopathic pulmonary fibrosis pathogenesis. Repetitive micro-injuries to the type II alveolar epithelial cells (type II AEC) in the predisposed individuals are leading to aberrant healing at the site of injury and resulting in the exaggerated production and accumulation of interstitial fibrosis. Ambient air pollutants, cigarette smoke, viral infections, occupational exposures and microaspirations act as triggers for the disease initiation in a genetically predisposed individual.

A body of evidence indicates that abnormal activation of alveolar epithelial cells (AEC) and fibroblasts in an aging lung plays a central role in the pathogenesis of IPF. The actual hypothesis of IPF pathogenesis points out the repetitive micro-injuries to the alveolar epithelium occurring in the predisposed individuals, in whom susceptibility is a consequence of the cellular senescence and accelerated epithelial cells aging, leading to aberrant healing at the site of injury and resulting in the exaggerated production and accumulation of interstitial fibrosis [3], see Figure 1. Type II AEC, responsible for the renovation of the alveolar epithelium, become “exhausted” and unable to restore normal lung architecture. Collagen and other components of extracellular matrix (ECM) replace normally structured alveoli, leading to irreversible loss of alveolar surface and impairment of gas transfer through the alveolar–capillary interface. Fibroblasts and myofibroblasts are effector cells that are direct sources of ECM components. Various growth factors and other pro-fibrotic cytokines are responsible for the formation of a pro-fibrotic microenvironment [3]. In the context of ambient air pollution as an important risk factor for IPF, several interrelated elements of the pathogenesis of lung fibrosis are of special interest. These include oxidative stress related to external (environmental) and internal (host-related) sources of reactive oxygen species (ROS), mitochondrial dysfunction and short telomeres as a cornerstone of accelerated aging of lung cells predisposing to ineffective healing, cellular senescence as a consequence of the latter, and, last but not least, a complex process of ECM remodeling, which includes mobilization of fibroblasts and myofibroblasts to the lung, different mechanisms responsible for “shifting” from physiological restoration of alveoli to uncontrolled scar formation, and deposition of ECM components [3,10,11,12,13].

#### 2.2.1. Oxidative Stress

The term “oxidative stress” describes the situation when excess of ROS production and/or antioxidant defense depletion results in molecular, cellular and tissue abnormalities. The term “oxidant-antioxidant imbalance” is also frequently used [14]. Oxidative stress is one of the most important mechanisms leading to fibrosis of various internal organs, including lungs. During summertime, photochemical reactions driven by sunlight lead to the conversion of organic compounds and oxides of nitrogen into photochemical oxidants, mainly O_3_. The reaction of nitric oxide (NO) and O_3_ leads to the generation of NO_2_. Under most atmospheric conditions NO and NO_2_ coexist as a mixture (NO_x_). These highly reactive substances contribute to the production of hydroxyl radical (OH·) or organic peroxy radicals [8]. The most reactive ROS, which is OH·, is produced from hydrogen peroxide (H_2_O_2_) in the reaction catalyzed by ferrous ions (Fe^2+^), or other transition metals, in the Fenton reaction [15]. This reaction may occur already on the surface of fine and ultrafine particles. Hydroxyl radical may also be produced by epithelial cells exposed to PM from superoxide anion [16]. Exposure to fine and ultrafine PM generates ROS-mediated oxidative stress in lung epithelium. For instance, exposure of cultured AEC to PM_2.5_ leads to generation of ROS and downregulation of nuclear factor erythroid 2-related factor 2 (Nrf-2), transcription factor responsible for synthesis of intracellular antioxidants, such as superoxide dismutase [17]. It is of note that the activation of Nrf2 signaling plays an essential role in preventing cells and tissues from injury induced by oxidative stress [18]. In lungs exposed to polluted air, both inflammatory cells and non-inflammatory cells (including AEC), may produce ROS by their intrinsic enzymes, like nicotinamide adenine dinucleotide phosphate oxidase (NADPH-oxidase) [19]. ROS can function as intermediary signaling molecules, and lead to the activation of tyrosine kinase receptor, mitogen-activated protein (MAP) kinase, nuclear factor-kappa B (NF-κB), and signal transducer and activator of transcription 1 (STAT-1). The consequence of these processes is activation of genes responsible for the production of various inflammatory cytokines of known pro-fibrotic properties, such as tumor necrosis factor-alpha (TNF-alpha) and interleukin (IL)-1 [20]. Another very important source of ROS in IPF pathogenesis and promotion of fibrosis are mitochondria. Mitochondrial dysfunction results in the generation of ROS, as the electron transport chain is uncoupled from proton pumping, and superoxide anion and H_2_O_2_ are released to the cytosol. Regardless of the sources, excessive generation of ROS may induce DNA damage, lipid peroxidation of cellular membrane lipids, p53 activation, cell cycle blockade and cell death via apoptosis or necrosis [21].

There are sufficient in vivo data confirming the oxidative stress-induced injury in the pathogenesis of IPF. Bleomycin-induced pulmonary fibrosis in mice and other animals is accompanied by ROS production, lipid peroxidation, DNA damage, and oxidation of proteins [22]. Moreover, exposure of mice with bleomycin-induced pulmonary fibrosis to PM_2.5_ from straw burning exacerbated lung inflammation and fibrosis and increased mortality [23]. Oxidized proteins, lipid peroxides, or carboxylated proteins have been detected in different biological materials (exhaled breath condensate, bronchoalveolar lavage fluid, or tissue samples) derived from IPF patients [24].

#### 2.2.2. Mitochondrial Dysfunction

Mitochondrial homeostasis and function is critical for the normal physiology of cells in diverse organ systems. Mitochondrial dysfunction, on the other hand, is a key player in the myriad of disease pathologies, including IPF [25,26,27]. Moreover, mitochondrial dysfunction is a recognized hallmark of aging, which plays a central role in IPF [28]. PM can induce mitochondrial toxicity with consequent overproduction of ROS by mitochondria, dysregulation of the electron transport chain, loss of mitochondrial membrane potential and impaired oxidative phosphorylation [29,30]. As a consequence, altered mitochondrial function leads to cell apoptosis and senescence, well known drivers of IPF [31]. Research data show that, apart from ROS generation, PM may induce AEC apoptosis by the interaction of p53 with mitochondrial apoptosis pathways [32,33]. Other altered function of mitochondria in IPF include decreased mitochondrial biogenesis, and impaired mitochondrial macroautophagy [34]. Mitochondrial dysfunction and metabolic reprogramming has been identified in different IPF lung cells (i.e., AEC, fibroblasts, and macrophages) increasing susceptibility to activation of profibrotic responses [28,34]. Accumulated research data are linking altered mitochondrial function with IPF [35,36,37,38,39,40,41,42,43].

#### 2.2.3. Telomere Shortening

Telomere attrition is associated with the pathogenesis of IPF [44]. Mutations in telomerase genes: telomeric RNA component (TERC) and telomerase reverse transcriptase (TERT), have been found in 8–15% of familial pulmonary fibrosis and 1–3% of sporadic IPF [45,46]. Shorter telomeres are frequently found in patients with IPF, even in the absence of TERC/TERT mutations [47]. It was found that telomere attrition is associated with long-term exposure to black carbon, the most toxic component of PM_2.5_, related to automobile traffic [48]. One of the most important inducing factors of telomere shortening is oxidative stress [49]. It has been proposed that telomeres can be considered as the cellular memories of exposure to oxidative stress and inflammation related to environmental exposure. The term “exposome” was proposed [50]. There are several reports proving telomere shortening in people chronically exposed to polluted air. For instance, in one study, traffic officers in Milan, Italy, had shorter leukocyte telomeres compared to age and sex-matched office workers, and within the study group of traffic officers, the telomere length was shorter in those who were exposed to more intensive road traffic [10]. Of note, as telomere length during in utero life and at birth might predict the overall life expectancy, exposure to air pollution during pregnancy might be of special importance for morbidity and mortality throughout adult life, predisposing to diseases such as IPF [50].

#### 2.2.4. Cellular Senescence

IPF is related to accelerated aging. Hallmarks of aging are genomic instability, telomere attrition and epigenetic alterations leading to altered mitochondrial functioning, and cellular senescence. Type II AEC are responsible for the renovation of injured lung epithelium. When type I cells are damaged, a subset of type II cells take over the task of stem cells and start to differentiate to type I AEC. IPF may be described as a disease of impaired alveolar healing due to dysfunctional type II AEC. Type II cells of the so-called “senescent phenotype” are unable to take-up this function. There are several interconnected factors responsible for this phenomenon, including embryonic developmental regulator signaling pathways (Wnt, Hedgehog etc.), expression of Fas-FasL pathway, Bcl-2, caspase-3 and other pro-apoptotic proteins, endoplasmic reticulum (ER) stress and impaired unfolded protein response (UPR), and mitochondrial dysfunction, leading to apoptosis [51,52]. The depletion of type II AEC, which also occurs in IPF, and the senescent phenotype trigger pro-fibrotic response, resulting in the influx and activation of myofibroblasts and excess production of collagen. Experimental studies have shown, that PM_2.5_ exposed type II alveolar epithelial A549 cells are prone to apoptosis, and this process is dependent on the production of ROS [53]. New data show that PM_2.5_–induced cell injury is mediated by oxidative stress and involves NF-κB induction of proinflammatory genes [54]. OGG1 (8-oxoguanine DNA glycosylase1), a DNA repair enzyme, plays a crucial role in protecting cells from oxidative damage and apoptosis induced by PM_2.5_. In a recent study, it was shown that this enzyme works through the inhibition of NF-κB signaling induced by oxidative stress [11].

#### 2.2.5. Extracellular Matrix Remodeling

Fibrosis is characterized by uncontrolled deposition of ECM components in the basement membrane and interstitial tissue as a result of the alveolar epithelium injury. Activated mesenchymal cells (myofibroblasts) are the direct sources of collagen and other ECM components. There are two main cellular sources of myofibroblasts considered important in the pathogenesis of IPF, particularly circulating fibrocytes—bone marrow derived cells, and epithelial-mesenchymal transition (EMT), the process during which fibroblasts and myofibroblasts are formed from epithelial cells in a specific pro-fibrotic microenvironment molded by pro-fibrotic inflammatory cytokines and growth factors. Transforming growth factor beta (TGF-beta), connective tissue growth factor (CTGF), fibroblast growth factor (FGF), platelet derived growth factor (PDGF), vascular endothelial growth factor (VEGF), and Th2 immune response cytokines, mainly IL-4, IL-13, are the main regulatory factors involved in this process in patients with IPF [12,55,56]. Different components of polluted air are responsible for increased production of these molecules, as shown in experimental studies. Titanium dioxide increases the levels of TGF-beta, TGF-alpha, and PDGF in rat tracheal explants [57]. It was also shown that urban ambient particles from Mexico City were able to induce PDGF receptors in myofibroblasts [58]. Moreover, PM_10_ from Mexico City increased protease activity in AEC by increasing expression of matrix metalloproteinases (MMP), mainly MMP-2 and MMP-9 [59]. PM_2.5_ exposure increased expression of TGF-beta1, alpha-smooth muscle actin and collagen type I in mice lung, and activation of TGF-beta/SMAD intracellular pathway was shown in cell lines [60]. It was also shown that cultured type II AEC become more susceptible to EMT after exposure to PM_2.5_ [13].

In summary, cumulative data from the experimental studies strengthen the notion, that different components of polluted air are responsible for initiation of several processes, which are crucial in IPF pathogenesis.

## 3. Impact of Air-Pollution on Disease Initiation and Health Outcomes in IPF

Apart from the genetic predispositions, numerous non-genetic risk factors for IPF have been identified. Older age, male sex, and cigarette smoking represent the most widely recognized [61], although microaspirations and gastroesophageal reflux disease, certain viruses, and occupational exposures (metal and wood dust) have also been suggested to increase the risk of IPF [61,62,63,64,65]. Despite notable research interest in air pollution relationship with respiratory diseases, very little is known on the short and long-term effects of air pollution in IPF. A recent systematic review providing supporting link between air pollution and fibrotic lung diseases included six studies on IPF and one study on hypersensitivity pneumonitis (HP) [66]. Below, we present the most current evidence on the association between ambient air pollution and IPF summarizing descriptively findings of 10 clinical studies identified as relevant for the scope of our topic.

### 3.1. Clinical Studies Linking Air Pollution with Initiation of IPF

A retrospective, longitudinal cohort study investigated, for the first time, the relationship between chronic exposure to air pollution, namely PM_10_, NO_2_, and O_3_ and the incidence of IPF. The study was undertaken in the Lombardy region of Northern Italy, using regional healthcare administrative databases analysis for the identification of the incident cases of IPF from 2005 to 2010. Exposures assessments included daily predictions of PM_10_ concentrations. Background and traffic monitoring stations were used for the measurements of NO_2_ and O_3_ concentrations. The study results showed that IPF incidence was not associated with the ambient air concentrations of PM_10_ and O_3_, but a significant impact of cold season NO_2_ concentration was found to affect the incidence of IPF. An increment of 10 µg/m^3^ in NO_2_ concentration was associated with a 7.93% increase in the incidence rate of IPF [67]. It is well known that NO_2_ is a surrogate of traffic-related exposure [68]; therefore, the study results provide a strong signal that traffic pollution could be involved in the initiation of the fibrotic process and identify a potentially modifiable risk factor for IPF [67]. A recent retrospective study aimed to identify environmental risk factors for IPF development [69]. Using the cross-analysis of the incident cases of the disease in the Catalan region, Spain, and estimated exposure to PM_2.5_ over the last decade, authors found a coincidence between IPF patient aggregation and PM_2.5_ concentration. Despite limitations resulting from the nature of that retrospective, observational study, its results suggest that environmental factors may add to development of IPF and should be considered for future prospective research on disease pathogenesis.

Another long-term large prospective study evaluated whether 10-year residence-specific exposure to ambient air pollution is associated with findings of interstitial lung abnormalities (ILAs) and high attenuation areas (HAA), which are two radiographic qualitative and quantitative measurements of subclinical ILDs in computed tomography [70]. Despite the aforementioned study has not assessed a direct relationship between air pollution and incidence of IPF, it should be noted that screening for ILAs might eventually provide a means for the early identification of IPF [71]. The authors of this study used sophisticated modeling to produce individual estimates of long-term exposure to air pollution (using residential history data) and investigated the effect of different lag times of exposure. The study results showed that ILAs were associated with long-term average exposure to NO_x_. The odds of ILAs increased 1.77 fold per 40 ppb increment in NO_x_ concentration, while ILAs were not associated with ambient PM_2.5_, NO_2_, or O_3_ concentrations. The study findings, for the first time, link the air pollution exposure with early subclinical evidence of ILDs, supporting the hypothesis that air pollution may contribute to the initiation of the pathogenic process in ILDs [70]. A more recent study of Framingham cohort participants evaluated associations of long-term exposure to traffic and ambient pollutants with odds of ILAs and their progression on repeated imaging [72]. Participants’ home addresses were geocoded and 5-year average exposures to PM_2.5_, elemental carbon (EC, a traffic-related PM_2.5_ component), and O_3_ were estimated. The study results revealed that higher long-term exposure to EC was associated with 1.27 (95% CI 1.04–1.55) times greater odds of ILAs, and 1.33 (95% CI 1.00–1.77) times greater odds of ILAs progression on sequential CT. These findings supporting an impact of traffic air pollution exposure, measured by ambient EC concentrations, on the risk of subclinical ILDs are in line with the previous findings of associations between ambient NO_2_ concentrations, a surrogate of traffic-related exposure, and incidence rate of IPF [67]. Thus, it can be speculated that traffic air pollution may be a trigger for the development of ILAs, which, in predisposed individuals combined with continued environmental exposures, may progress to clinically apparent IPF. Future, long-term prospective studies are needed to clarify that hypothesis.

### 3.2. Clinical Studies Linking Air Pollution with the Short and Long-Term Health Outcomes in IPF

A small prospective study evaluated the relationship between air pollution exposure, lung function, and dyspnea in patients with IPF. Weekly mean air pollution exposures for ground-level O_3_, NO_2_, PM_2.5_, and PM_10_ were estimated based on the participants’ place of living [73]. The study results indicated that increased exposures to NO_2_, PM_2.5_, and PM_10_ were associated with lower forced vital capacity (FVC) in studied patients with IPF, suggesting that air pollution may impact disease severity in some individuals. Despite a small sample size and a relatively short period of follow-up, this study provides new insight into the impact of air pollution on health outcomes in IPF, suggesting that an individual’s disease may be more severe due to environmental exposure to air pollution [73]. Although the latter study did not find any relation between air pollution exposure and progression of the disease as measured by the rate of decline of FVC in the studied cohort, this has been reported by a recent retrospective study held at the single university referral center in the USA [74]. The authors analyzed whether increased exposure to air pollution in the form of ambient PM_2.5_ and PM_10_ would be associated with an accelerated rate of decline of pulmonary function in IPF. Ambient exposures to PM were estimated using daily mean concentration levels of PM collected at the air quality monitoring stations nearest the subjects’ home addresses (geocoding). The study results pointed out a significant relationship between exposure to ambient PM_10_ and the rate of decline in FVC. The authors found that each 5 μg/m^3^ increase in PM_10_ concentration was corresponding to an additional 46 mL decline in FVC per year. This study clearly demonstrated an association between exposure to ambient PM_10_ and widely accepted for the clinical practice indicator of the disease progression in IPF [74].

A recent, retrospective study aimed to assess whether acute increases in air pollution are risk factors for hospitalization of patients with IPF [75]. This research was based on the longitudinal analysis of healthcare administrative databases and air pollution exposure data in Santiago, Chile. The study results indicated that hospital admissions of patients with IPF were significantly higher on, or closely following, days of higher air pollution and were less frequent on days when pollutants concentrations were lower. In the overall cohort, two-pollutant models pointed out that the most robust associations were with NO_2_ and PM_10_, suggesting their strongest independent impact on the hospitalization risks in IPF.

The natural history of IPF is heterogeneous and unpredictable, ranging from a stable decline of lung function to rapid progression and death. Some patients may also develop an acute exacerbation of IPF (AE-IPF) with a fatal outcome. AE-IPF may be categorized as idiopathic or triggered by various known insults. Such an episode has a poor prognosis with a median survival of patients who experience AE-IPF of 3–4 months and high in-hospital mortality due to respiratory failure reaching 50% [76]. Two previous studies evaluated the association of air pollution exposure with AE-IPF. The first one was undertaken as a prospective, longitudinal cohort study including 436 Korean patients with IPF [77]. The authors analyzed whether ambient air pollution exposure to O_3_, NO_2_, PM_10_, SO_2_, and CO is linked to AE-IPF. Air pollution data for each of the five pollutants were measured during a study period using a dedicated geocoded monitoring system throughout Korea. The study results indicated that the risk of AE-IPF was significantly associated with an increased mean level of O_3_ (hazard ratio [HR] 1.57; 95% CI 1.09–2.24) and NO_2_ (HR 1.41; 95% CI 1.04–1.91) within the preceding 6 weeks. No associations were found for PM_10_, SO_2_, and CO with the risk of AE-IPF. Air pollution exposure was not found to increase overall mortality in the studied cohort. Taking into account the above study finding, it is plausible that at least part of idiopathic AE-IPF may be caused by exposure to ambient air pollution, therefore not being true idiopathic [78]. A recent study demonstrated that exposure to benzo[a]pyrene, which is one of the most studied ambient air PAHs, worsens murine fibrosis and myofibroblast activation via reduction of protein G-protein-coupled receptor family C group 5 type A (GPRC5A) in the damaged epithelium [79]. Another study using similar method of geocoded evaluation of exposure to ambient air pollution was performed on the French cohort participating in the national multicenter prospective study on the natural history of IPF conducted over a period of 7 years [80]. Ambient air pollutants exposures, namely NO_2_, O_3_, PM_2.5_, and PM_10_, were analyzed and linked to AE-IPF, disease progression, and death. Noted results pointed out that AE-IPF events were significantly associated with a higher mean concentration of O_3_ within the preceding 6 weeks with an HR of 1.47 (95% CI 1.13–1.92) per 10 µg/m^3^. No association was observed between AE-IPF and NO_2_ and PM_2.5_ or PM_10_. The finding of a significant association of ambient exposure to O_3_ and increased risk of AE-IPF in the French cohort is in the concordance of a similar finding in the Korean cohort of patients with IPF [77]. Such consistency of the results obtained, despite the relatively small sample sizes of these two studies, supports the validity of the finding that O_3_ may impact the risk of AE-IPF. Therefore, it may be assumed that reductions in ambient air pollution exposure may decrease the risk of AE-IPF and improve the morbidity associated with IPF. Evaluation of the long-term effect of the cumulative air pollution exposure did not show any influence of the cumulative concentration of studied pollutants on disease progression, but it is of note that mortality was significantly associated with cumulative concentration of PM_2.5_ with an HR of 7.93 (95% CI 2.93–21.33) per 10 μg/m^3^ and PM_10_ with an HR of 2.01 (95% CI 1.07 to 3.77) per 10 μg/m^3^ [80]. Another recent, retrospective, observational study undertaken in 1114 patients with IPF from the Korean referral center aimed to evaluate the impact of exposure to PM_10_ and NO_2_ on mortality [81]. Exposure assessments were estimated using geocoding. The authors did not confirm the latter study findings of an association between PM_10_ exposure and mortality [80], but their results indicated a significant association between long-term ambient NO_2_ exposure and mortality in patients with IPF. It was estimated that a 10-ppb increase in NO_2_ concentration was associated with a 17% increase in mortality (HR 1.172; 95% CI: 1.030–1.344). Both studies add empirical evidence of the negative impact of the cumulative air pollution exposure on the long-term health outcomes in IPF [80,81].

**Figure 2:**
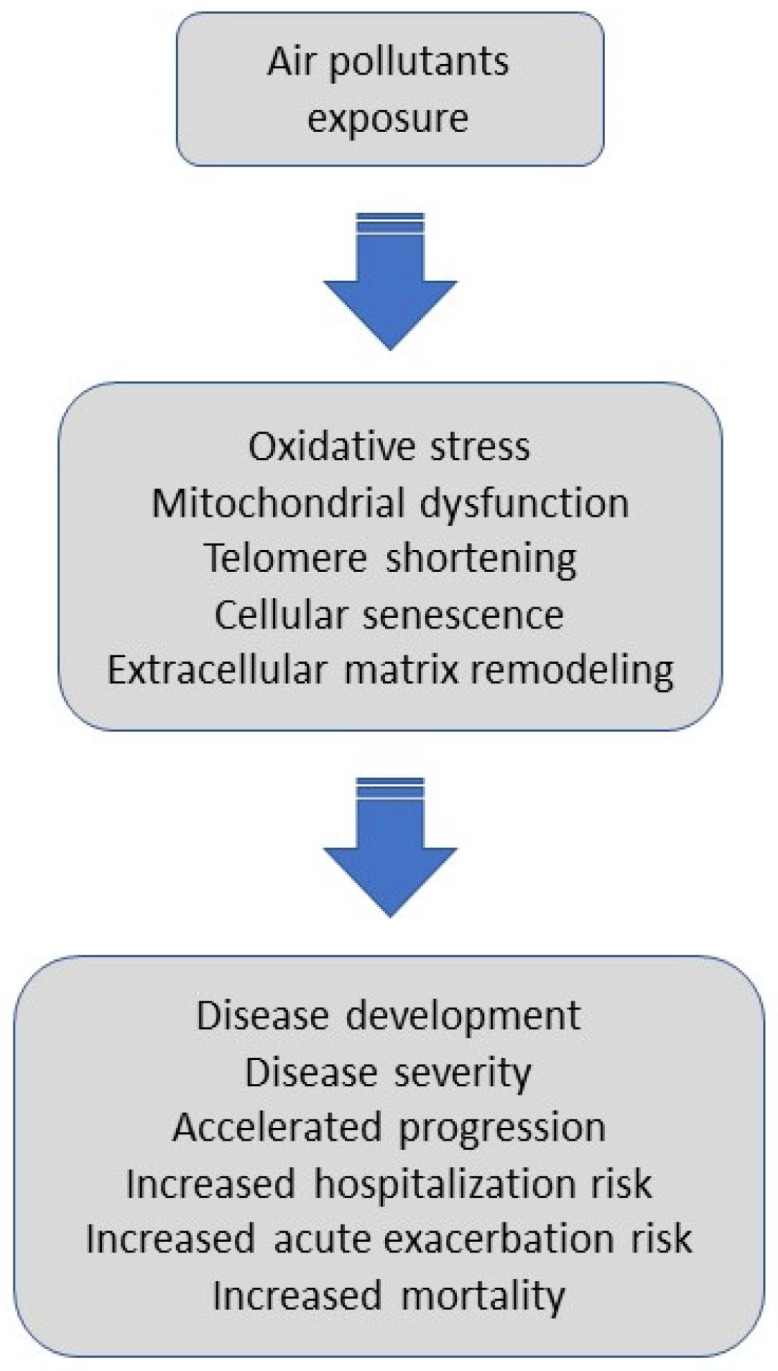
Potential mechanisms triggered by air pollutants and their impact on outcomes in IPF. Air pollutants exposure, via oxidative stress, mitochondrial dysfunction, telomere shortening, cellular senescence, and extracellular matrix remodeling might promote disease development, affect an individual’s disease severity, accelerate progression, trigger hospitalization or acute exacerbation, and increase overall mortality in patients with IPF.

The summary of the literature reviewed linking air-pollution exposure with disease initiation and health outcomes in IPF is shown in Table 1. Potential mechanisms triggered by air pollutants and their impact on outcomes in IPF are illustrated in Figure 2.

Available studies linking air pollution exposure with the initiation of disease and health outcomes in IPF are not free from inherent methodological limitations, which warrant cautious interpretation of their results. The estimations of air pollution exposures were based on aggregated data, geocoding, or used other existing data sources. This approach limits the precision with which the true exposures can be ascertained. Moreover, poor control for confounding related to unaccounted environmental or subject-specific factors (e.g., smoking, occupational exposure), as well as retrospective medical database analyses, which are subjected to the quality of IPF coding, may also influence research findings.

Despite the aforementioned limitations, findings linking air pollution and IPF have important clinical implications as identification of a potentially modifiable risk factor and its elimination or avoidance can improve short and long-term health outcomes in IPF.

To summarize, accumulating evidence on associations between ambient air pollution and IPF strengthens the view that air pollution exposure should be considered as an important risk factor in IPF. Future large and prospective, subject-specific, rather than aggregated data studies, accounting for more accurate individual subject’s exposures in the place of living are needed to corroborate previous findings.

## 4. Conclusions

A growing body of literature supports the causative link between air pollution and IPF. Ambient air pollutants through several potential mechanisms may contribute to disease initiation, impact an individual’s disease severity, accelerate progression, trigger hospitalization and acute exacerbation, as well as affect mortality. Our current understanding of the risks and health effects of air pollution exposure in IPF is expanding but is far from complete. The exposure assessment is the major challenge of environmental epidemiology applied to air pollution research. Thus, comprehensive accumulated environmental exposure assessments at the individual patient’s level and their linkage to the pathogenesis and course of IPF should become a priority in future research. Identification of the risk and approach towards its elimination or avoidance may result in meaningful benefits for the patients and global public health.

## Figures and Tables

**Table 1:** Summary of studies linking air-pollution exposure with disease initiation and health outcomes in IPF.

First Author, Publication Year [Reference]	Study Design	Aims of Study	Study Population	Study Findings
Conti S, 2018 [67]	A longitudinal retrospective cohort study	Association between long-term air pollution exposures and IPF incidence	2090 incident IPF cases in Lombardy, Italy	An increment of 10 µg/m^3^ in NO_2_ concentration was associated with a 7.93% increase in the incidence rate of IPF. Traffic-related pollution could be involved in the initiation of the fibrotic process in IPF.
Shull JG, 2020 [69]	A retrospective cohort study	Cross-analysis of geographic regions of IPF cases and mapping of PM_2.5_ concentration	379 patients with IPF from the registry in the Catalan region, Spain	The prevalence of IPF was higher in areas of elevated PM_2.5_ concentration. Certain areas with elevated air pollutants may be deserving greater analysis for screening of IPF.
Sack C, 2017 [70]	A prospective cohort study	Association between ambient air pollution and ILAs	2671 participants of the Multi-Ethnic Study on Atherosclerosis (MESA), USA	Long-term exposure to ambient NO_x_ was associated with higher prevalence of ILAs. The odds of ILAs increased 1.77-fold per 40 ppb increment in NO_x_. Air pollution exposures were associated with subclinical ILDs.
Rice MB, 2019 [72]	A longitudinal prospective cohort study	Association between long-term exposure to traffic and ambient pollutants and incidence of ILAs and their progression	2618 Framingham study participants, USA	Higher 5-year average exposure to elemental carbon (a traffic-related PM_2.5_ component) was associated with 1.27 times greater odds of ILAs, and 1.33 times greater odds of ILAs progression on sequential imaging. Long-term exposure to traffic-related pollution may lead to interstitial remodelling.
Johansson KA, 2018 [73]	A longitudinal prospective cohort study	Association between air pollution exposure and lung function in IPF	25 patients with IPF, USA	Increased average exposures to NO_2_, PM_2.5_ and PM_10_ were associated with lower FVC in patients with IPF. Air pollution may have an influence on disease severity in IPF.
Winterbottom CJ, 2018 [74]	A retrospective cohort study	Association between exposures to PM_2.5_ and PM_10_ and lung function decline in IPF	135 patients with IPF, USA	A significant association between PM_10_ levels and the rate of decline in FVC during the study period was noted, with each mg/m^3^ increase in PM_10_ corresponding with an additional 46 mL decline in FVC yearly. Air pollution exposure was associated with progression of IPF.
Dales R, 2020 [75]	A longitudinal retrospective cohort study	Association between acute increases in air pollution exposures and risk of hospitalization in patients with IPF	Patients hospitalized with a primary diagnosis of IPF identified in the healthcare databases of the province of Santiago, Chile	Hospital admissions of patients with IPF were significantly higher on, or closely following days of higher air pollution. The most robust associations were noted for NO_2_ and PM_10._ Acute increases in air pollution are a risk factor for hospitalization of patients with IPF.
Johansson KA, 2014 [77]	A longitudinal prospective cohort study	Association between air pollution exposure and AE-IPF	436 patients with IPF, South Korea	The risk of AE-IPF was significantly associated with an increased mean level of O_3_ (57% increased risk) and NO_2_ (41% increased risk) within the preceding 6 weeks. Increased exposure to air pollutants contributes to the development of AE-IPF.
Sesé L, 2018 [80]	A longitudinal prospective cohort study	Impact of air pollution exposures on the natural history of IPF	192 patients with IPF, France	The risk of AE-IPF events was significantly associated with a higher mean concentration of O_3_ within the preceding 6 weeks with 47% increased risk per 10 µg/m^3^. Mortality was significantly associated with cumulative concentration of PM_2.5_ and PM_10._ Air pollution has a negative impact on the short and long-term outcomes in IPF.
Yoon HY, 2020 [81]	A longitudinal retrospective cohort study	Association between air pollution exposure and mortality in IPF	1114 patients with IPF, South Korea	A 10-ppb increase in NO_2_ concentration was associated with a 17% increase in mortality of patients with IPF. Air pollution exposure can increase mortality in IPF.

## Abbreviations

IPF	idiopathic pulmonary fibrosis
AE-IPF	acute exacerbation of idiopathic pulmonary fibrosis
FVC	forced vital capacity
ILAs	interstitial lung abnormalities
ILDs	interstitial lung diseases
NO_2_	nitric dioxide
NOx	nitric oxides
PM_2.5_	particulate matter < 2.5 μm
PM_10_	particulate matter < 10 μm
O_3_	ozone.
